# TERT mutations-associated alterations in clinical characteristics, immune environment and therapy response in glioblastomas

**DOI:** 10.1007/s12672-023-00760-w

**Published:** 2023-08-11

**Authors:** Feng Tang, Xi Chen, Jin-Sheng Liu, Zhen-Yuan Liu, Jin-Zhou Yang, Ze-Fen Wang, Zhi-Qiang Li

**Affiliations:** 1 Brain Glioma Center, Department of Neurosurgery, Zhongnan Hospital of Wuhan University, Wuhan, Hubei China; 2 Department of Physiology, Wuhan University School of Basic Medical Sciences, Wuhan, Hubei China

**Keywords:** TERT, Glioblastomas, Immune microenvironment, Immunotherapy

## Abstract

**Objective TERT:**

is the most frequently mutated gene in adult glioblastomas (GBMs) defined by the 2021 World Health Organization classification system. The present study aims to explore differences in clinical characteristics and immune microenvironment between TERT mutant and wild-type GBM.

**Methods:**

Three GBM-related cohorts consisting of 205 GBM patients in our cohort, 463 GBM patients without immune checkpoint inhibitor(ICI) therapy and 1465 tumour patients (including 92 GBM cases) receiving ICI treatment in the MSK cohort were included. Retrospective analysis and immunohistochemistry assay were used for investigating the local (including tumour cells, local immune cells, and seizures) and systemic (including circulating immune cells, coagulation-related functions, and prognosis) effects of TERT mutations. Besides, differences in genetic alterations and immunotherapy responses between TERT mutant and wild-type GBMs were also explored.

**Results:**

We found that TERT mutant and wild-type GBMs possessed similar initial clinic symptoms, circulating immune microenvironment and immunotherapy response. With respect to that in TERT wild-type GBMs, mutations in TERT resulted in higher levels of tumour-infiltrating neutrophils, prolonged coagulation time, worse chemotherapy response and poorer overall survival.

**Conclusion:**

Mutations in TERT alter the local immune environment and decrease the sensitivity of GBM to chemotherapy.

**Supplementary Information:**

The online version contains supplementary material available at 10.1007/s12672-023-00760-w.

## Introduction

Gliomas represent the most common malignant tumour in the central nervous system (CNS) [1]. The annual incidence rate of gliomas in China is 5–8/100,000, and the 5-year mortality rate is only second to pancreatic cancer and lung cancer in general tumours [2]. In the 2016 World Health Organization (WHO) classification of CNS tumors, adult gliomas are divided into isocitrate dehydrogenase (IDH) wild-type and mutant lower-grade gliomas (LGGs) with or without 1p/19q-codeletion, and IDH mutant and wild-type glioblastomas (GBMs) [3]. According to the newest 2021 WHO classification of CNS tumours, IDH wild-type LGGs with telomerase reverse tranase (TERT) mutation and IDH wild-type GBMs in the 2016 classification were divided into GBMs (GBMs) [4].

Telomerase, a ribonucleoprotein polymerase consisting of an RNA component and a protein component TERT, is essential for maintaining the replication of chromosome termini in most eukaryotes [5–7]. In postnatal somatic cells, telomerase expression is normally repressed, which leads to the progressive shortening of telomeres and cellular senescence [8]. However, mutations in the TERT promoter were found in most types of tumours, which increased the expression of TERT and enhanced telomerase activity [9]. Mutations in TERT promoter endow tumour cells with the characteristic of “immortality”, allowing them to grow unrestricted [7, 9]. Dysregulated TERT participate in the occurrence of breast cancer, lung cancer, thyroid cancer, liver cancer, melanoma and other tumours, and mutations in TERT promoter are closely related to the occurrence, development and prognosis of these tumours [7, 10, 11]. In addition to neoplasm cells, mutant TERT could also participate in regulating immune cell infiltration and act as a prognostic factor in patients treated with an immune checkpoint inhibitor(ICI) [12, 13]. In IDH mutant gliomas, patients with TERT mutation exhibited better prognosis than those with wild-type. In contrast, TERT mutation is an indicator of poor prognosis in IDH wild-type gliomas [14]. However, the differences in genetic and clinical features between TERT mutant and wild-type GBMs remain to be illustrated.

In the present study, 205 GBM patients in our cohort, 463 GBM patients without immune checkpoint inhibitor(ICI) therapy and 1465 tumour patients (including 92 GBM cases) receiving ICI treatment in the MSK cohort were included. The clinical data of 118 patients with TERT mutant GBMs and 87 patients with TERT wild-type GBMs was retrospectively reviewed, and the local (including tumour cells, tumour-infiltrating immune cells, and seizures) and systemic (circulating immune cells, coagulation-related functions, and prognosis) characteristics were examined. Data from the MSK cohort were used for exploring differences in genetic alterations and immunotherapy responses between TERT mutant and wild-type GBMs. Hopefully, this study will contribute to understanding how TERT mutant GBMs develop, as well as future chemo- and immunotherapies.

## Materials and methods

### Data collection

Clinical and pathological data on 205 adult patients with GBMs were retrospectively collected from 2016 to 2022 in the Department of Neurosurgery at Zhongnan Hospital of Wuhan University (Table 1). Mutant or amplified or methylated status of TERT, EGFR and MGMT were evaluated by next generation sequencing. A systemic effect of TERT mutations on the circulation of blood of 205 GBM patients was investigated by collecting peripheral blood routine tests and coagulation function tests prior to surgery. Besides, an immunohistochemistry assay was performed on 58 GBM tissue samples from our hospital to examine the differences between TERT mutant and wild-type GBMs in the local immune system. No other malignant tumours or chronic inflammatory diseases (including infections and autoimmune diseases) were present in any of the patients included in this study. This study was approved by the Ethics Committee of Zhongnan Hospital of Wuhan University (No. 2,019,048).

**Table 1:** Characteristics of 205 patients with GBMs in our cohort

Variable	Value
No. of patients	205
Sex	
Male	124(60.5%)
Female	81 (39.5%)
Age at diagnosis, yrs	
Median	58 (20–81)
≤ 58	104(50.7%)
> 58	101(49.3%)
Location	
Frontal lobe	48(23.4%)
Temporal lobe	54(26.4%)
Parietal lobe	15(7.3%)
Occipital lobe	8(3.9%)
Multiple lobes	49(23.9%)
Other	31(15.1%)
Initial clinic symptoms	
Dizzy or headache	97(47.3%)
Limb asthenia	23(11.2%)
Cognitive impairment	39(19.0%)
Seizures	29(14.2%)
Other	17(8.3%)
TERT mutation	
Yes	118(57.6%)
No	87(42.4%)

Genomic data from 463 GBMs patients not treated by ICI in the MSK cohort were collected to investigate the differences in genetic alterations between TERT mutant and wild-type GBMs. Besides, we also collected data from 1465 tumour patients receiving ICI treatment consisting of seven types of tumours to explore the effects of TERT mutations on ICI response in a pan-cancer analysis (https://www.cbioportal.org/datasets) [15].

### Immunohistochemistry staining

To stain 58 GBM patients (29 cases with TERT mutations, 29 cases with wild-type mutations), a tissue microarray containing 58 samples was used. Briefly, after paraffin removal and antigen repair, GBMs slices were sealed with goat serum for 30 min at room temperature. After that, the slices were incubated with corresponding antibodies: CD4 (1:200, 4B12, Dako); CD8 (1:200, C144B, Dako); CD20 (1:200, L26, Leica); CD45 (1:200, 2B11, Dako); CD68 (1:200, KP1, Dako); CD11b (1:200, #49,420, CST) overnight at 4 °C. On the following day, the slices were incubated with a secondary antibody for 1 h at 37 °C, followed by 5–10 min incubation with a DAB solution. As a final step, images were captured with a microscope imaging system (Olympus, Japan).

### Statistical analysis

SPSS 23.0 and GraphPad Prism 8.0 software were applied for statistical analysis in the study. An initial assessment of the distribution of the variables was performed using the Shapiro-Wilk test. In the case of normal data distribution, the Student’s t-test was applied, otherwise, the Mann-Whitney test was performed. The chi-square test or Fisher's exact test was used for the comparison of categorical variables. A *P* < 0.05 was considered statistically significant.

## Results

### Genetic alterations in adult IDH wild-type gliomas

**Fig. 1:**
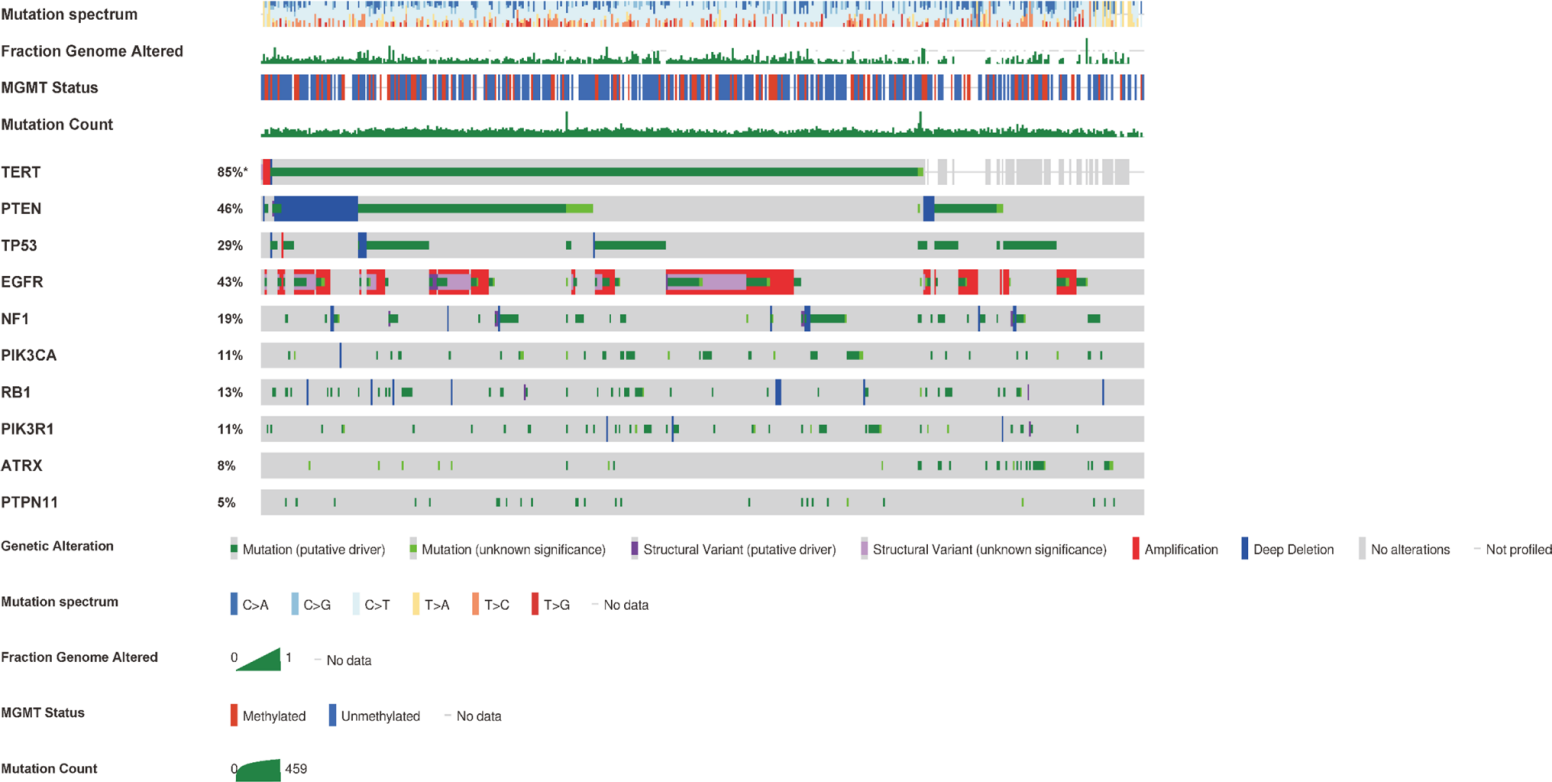
Oncoprint of gene alterations in IDH wild-type gliomas

In the 2016 WHO classification of CNS tumours, adult gliomas were divided into IDH mutant and wild-type gliomas. Compared to IDH mutant gliomas, patients with IDH wild-type exhibited poorer prognosis in the MSK cohort, which was consistent with previous findings (Supplematary Fig. 1) [16]. Genetic evolution is at the center of glioma progression [17]. In IDH wild-type gliomas, the frequency of genetic alterations in genes *TERT* (85%), *PTEN* (46%), *TP53* (29%), *EGFR* (43%), *NF1* (19%), *RB1* (13%), *PIK3CA* (11%), *PIK3R1* (11%), *ATRX* (8%), and *PTPN11* (5%) were the top-10 highest (Fig. 1). Among these genes, alterations in TERT owned the highest frequency and we, therefore, focused on exploring the differences in characteristics between TERT mutant and wild-type gliomas.

Among IDH wild-type gliomas, GBMs and TERT mutant LGGs were divided into GBMs in the 2021 WHO classification of CNS tumours. According to the 2021 classification, a total of 463 GBMs were included in the current study and mutation frequency in *TERT* (77%) was still the highest in the MSK cohort (Table 2). We then explored differences in genetic alterations between TERT mutant and wild-type GBMs. As shown in Table 2, in contrast to wild-type GBMs, TERT mutant GBMs showed a lower frequency of TP53 mutations (TERT mutant vs. wild-type: 26.0% vs. 37.1%, *P* = 0.026). However, the frequency of PTEN, EGFR, and NF1 mutations was similar between the two subtypes (Table 2).

**Table 2:** Difference in characteristics between TERT mutant and wild-type gliomas in MSK glioma cohort

Variable	TERT wild-type	TERT mutation	P-value
No. of patients	105	358	
Sex			0.168
Male	72(68.6%)	219(61.2%)	
Female	33(31.4%)	139(38.8%)	
Age			0.057
≤ 58	58(55.2%)	160(44.7%)	
> 58	47(44.8%)	198(55.3%)	
PTEN mutation			0.778
Yes	38(36.2%)	135(37.7%)	
No	67(63.8%)	223(62.3%)	
TP53 mutation			0.026
Yes	39(37.1%)	93(26.0%)	
No	66(62.9%)	265(74.0%)	
EGFR mutation			0.350
Yes	19(18.1%)	80(22.3%)	
No	86(81.9%)	278(77.7%)	
NF1 mutation			0.586
Yes	20(19.0%)	60(16.8%)	
No	85(81.0%)	298(83.2%)	
MGMT methylation			0.606
Hyper-methylation	23(32.4%)	84(29.3%)	
Hypo-methylation	48(67.6%)	203(71.7%)	

### Clinicopathological differences between TERT mutant and wild-type GBMs

Our cohort of adult GBMs was examined for clinicopathological differences between TERT mutant and wild-type cases. A total of 205 GBMs in our cohort were included, of these patients, 118 (57.6%) were TERT mutant and 87 (42.4%) were TERT wild-type GBMs (Table 1). No differences in sex and age were observed between the two subtypes of GBMs in both our cohort or the MSK cohort (Tables 2 and 3). Both TERT mutant and wild-type GBMs frequently occurred in the frontal lobe, temporal lobe and multiple lobes (Table 3). The initial clinic symptoms of the two subtypes of GBM patients were also similar. Although the incidence of epilepsy in TERT mutant GBM patients was slightly higher than that in wild-type patients, it did not reach statistical significance (Table 3). The incidence of EGFR amplification and low Ki67 index between TERT mutant and wild-type GBMs was also no different, indicating a similar proliferative capacity of the TERT mutant and wild-type glioma cells. In addition, the frequency of MGMT methylation between the two subtypes of GBMs was also similar (Table 3).

**Table 3:** Difference in characteristics between TERT mutant and wild-type gliomas in our cohort

Variable	TERT wild-type	TERT mutation	P-value
No. of patients	87	118	
Sex			0.448
Male	50(57.5%)	74(62.7%)	
Female	37(42.5%)	44(37.3%)	
Age			0.418
≤ 58	47(54.0%)	57(48.3%)	
> 58	40(46.0%)	61(51.7%)	
Location			0.246
Frontal lobe	20(23.0%)	28(23.7%)	
Temporal lobe	18(20.7%)	36(30.5%)	
Parietal lobe	8(9.2%)	7(5.9%)	
Occipital lobe	2(2.3%)	6(5.1%)	
Multiple lobes	21(24.1%)	28(23.7%)	
Other	18(20.7%)	13(11.1%)	
Initial clinic symptoms			0.480
Dizzy or headache	42(48.3%)	55(46.6%)	
Limb asthenia	10(11.5%)	13(11.0%)	
Cognitive impairment	16(18.4%)	23(19.5%)	
Seizures	9(10.3%)	20(16.9%)	
Other	10(11.5%)	7(8.0%)	
EGFR amplification			0.382
Yes	29(33.3%)	47(39.8%)	
No	58(66.7%)	71(60.2%)	
MGMT methylation			0.104
Hyper-methylation	35(40.2%)	61(51.7%)	
Hypo-methylation	52(59.8%)	57(48.3%)	
Ki67			0.356
Low (≤ 10%)	12(13.8%)	22(18.6%)	
High (> 10%)	75(86.2%)	96(81.4%)	

### Mutant TERT GBMs exhibit more tumour-infiltrating of neutrophils

**Fig. 2:**
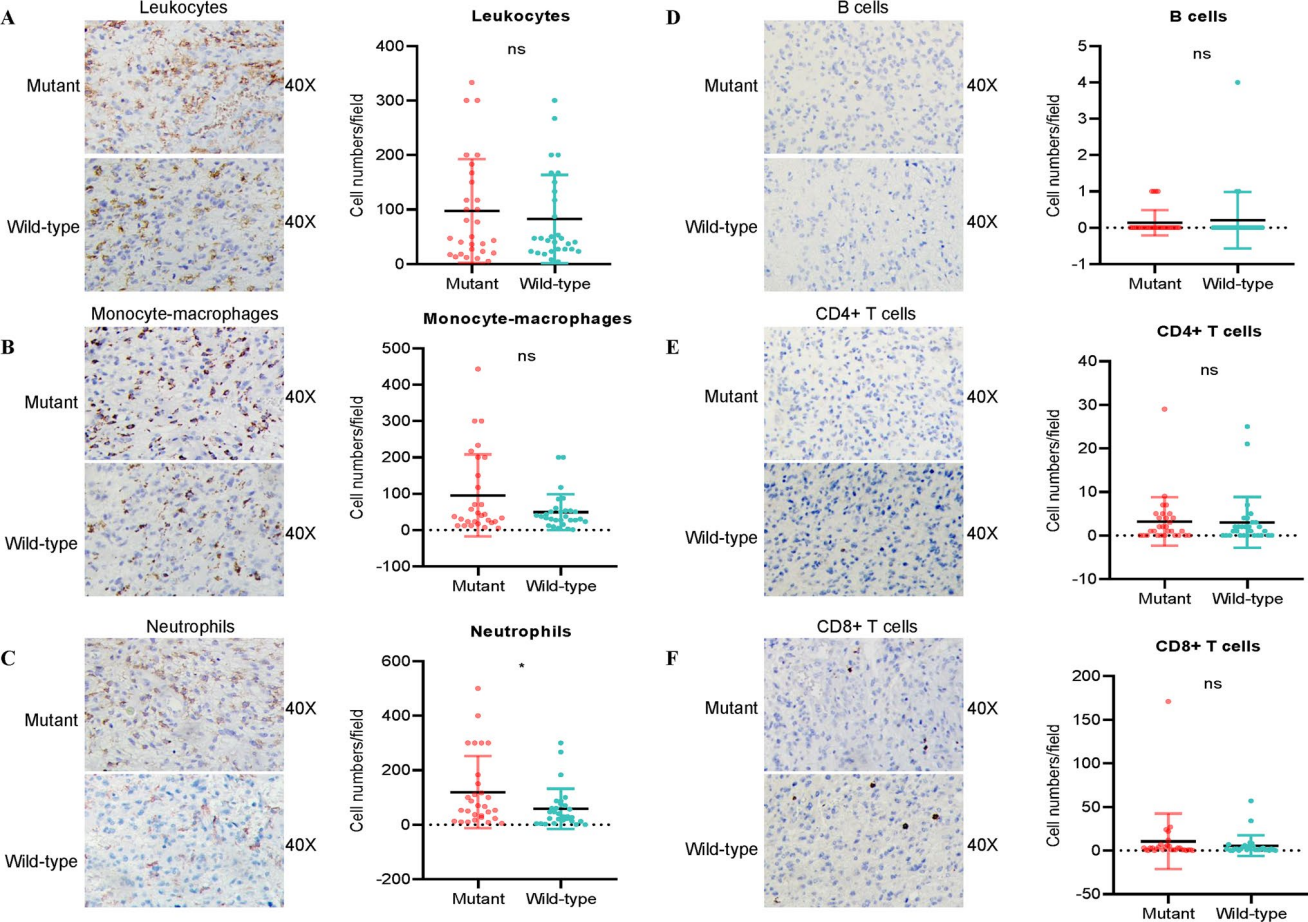
Dfference in tumour-infiltrating immune cells between TERT mutant and wild-type GBMs based on the results of lHC assay in our cohort. **A**-**F**. GBMs patients with TERT mutations harbour higher levels of neutrophils (**C**), but similar levels of other types of tumour-infitrating immune cells （leukocytes, monocyte-macrophages, B cells，CD4+ T cells and CD8+ T cells, **A**, **B**, **D**, **E**, **F**）compared to TERT wild-type. ^*^P < 0.05, ns means *P* > 0.05 compared to TERT wild-type

Our previous study found that mutations in IDH resulted in different local immune microenvironments [18]. Moreover, we conducted an IHC assay on GBM samples in our cohort to investigate local immune cell infiltration. As shown in Fig. 2A–C, although there was no difference in total leukocytes and macrophages in the local immune microenvironment between TERT mutant and wild-type GBMs, neutrophil numbers in TERT mutant GBMs were higher with respect to that in wild-type GBMs. In contrast, no difference was observed in levels of tumour-infiltrating B cells, CD4 + and CD8 + T cells between the two types of gliomas (Fig. 2D–F).

### Differences in the circulating immune microenvironment between TERT mutant and wild-type GBMs

**Fig. 3:**
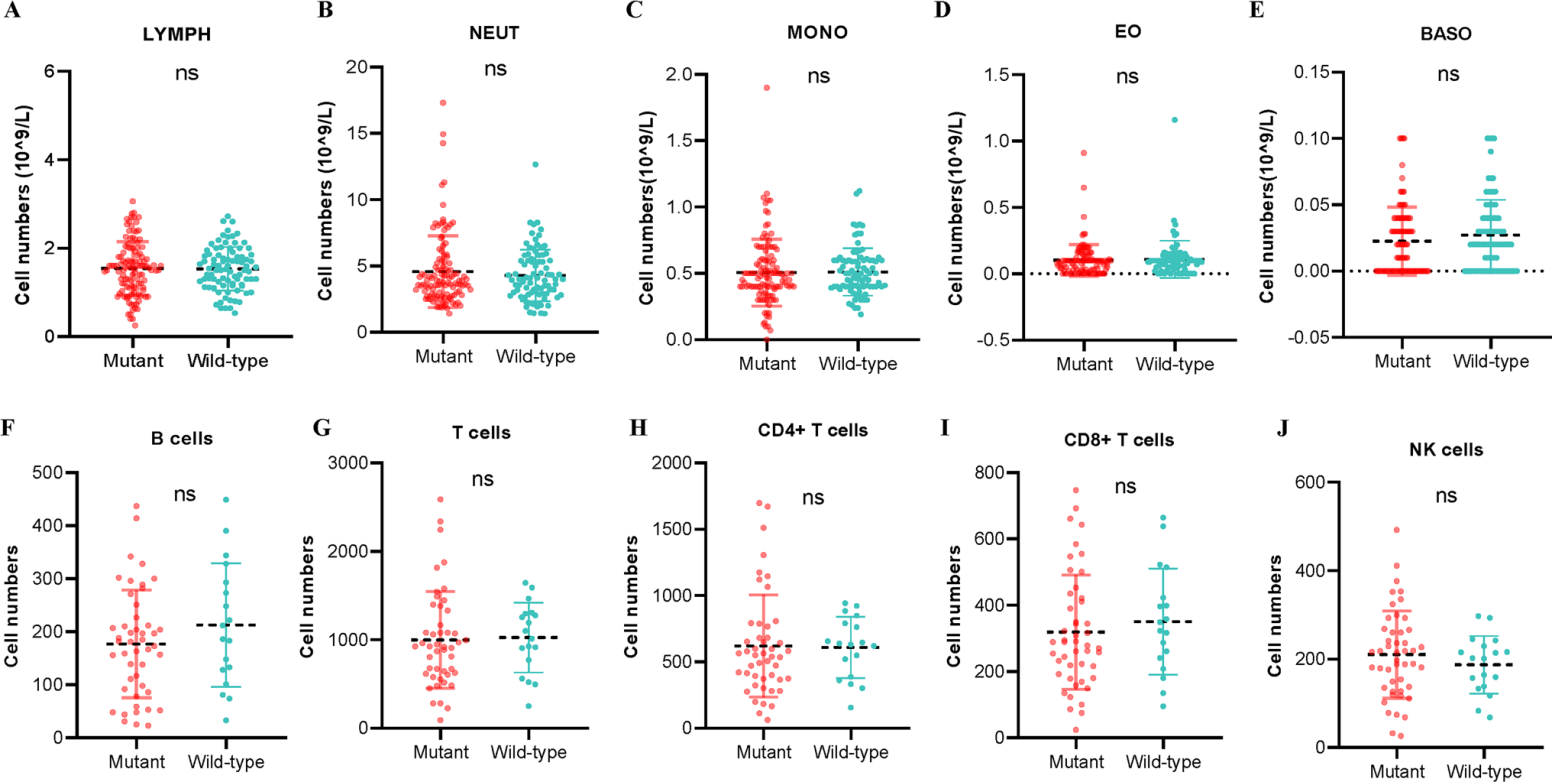
Mutant TERT did no effect on circulating immune cells of GBM patients based on peripheral blood data from our cohort.** A**-**J**. TERT mutant and wild-type GBM patients with own similar levels of circulating immune cells. ns means *P* > 0.05 compared to IDH wild-type

First, the median values of the parameters in 205 GBM patients’ routine blood tests were compared. As shown in Fig. 3A–E, the count of monocytes(MONO), neutrophils(NEUT), eosinophils(EO), basophils(BASO) and total lymphocytes(LYMPH) were comparable between patients with TERT wild-type and mutant GBMs. In addition, we investigated the lymphocyte subtypes (CD4 + T cells, CD8 + T cells, B cells, and NK cells) in GBM patients with TERT wild-types and mutations. Similarly, no difference was observed in CD4 + T cells, CD8 + T cells, B cells, or NK cells between TERT mutant and wild-type GBMs(Fig. 3F–J). Inflammatory markers were also examined in these two types of GBMs. However, we still did not observe differences in any types of inflammatory markers between TERT mutant and wild-type GBMs (Supplementary Fig. 2). These results suggested that mutations in TERT were unlikely to affect the circulating immune microenvironment.

### Mutant TERT patients exhibited prolonged coagulation

**Fig. 4:**
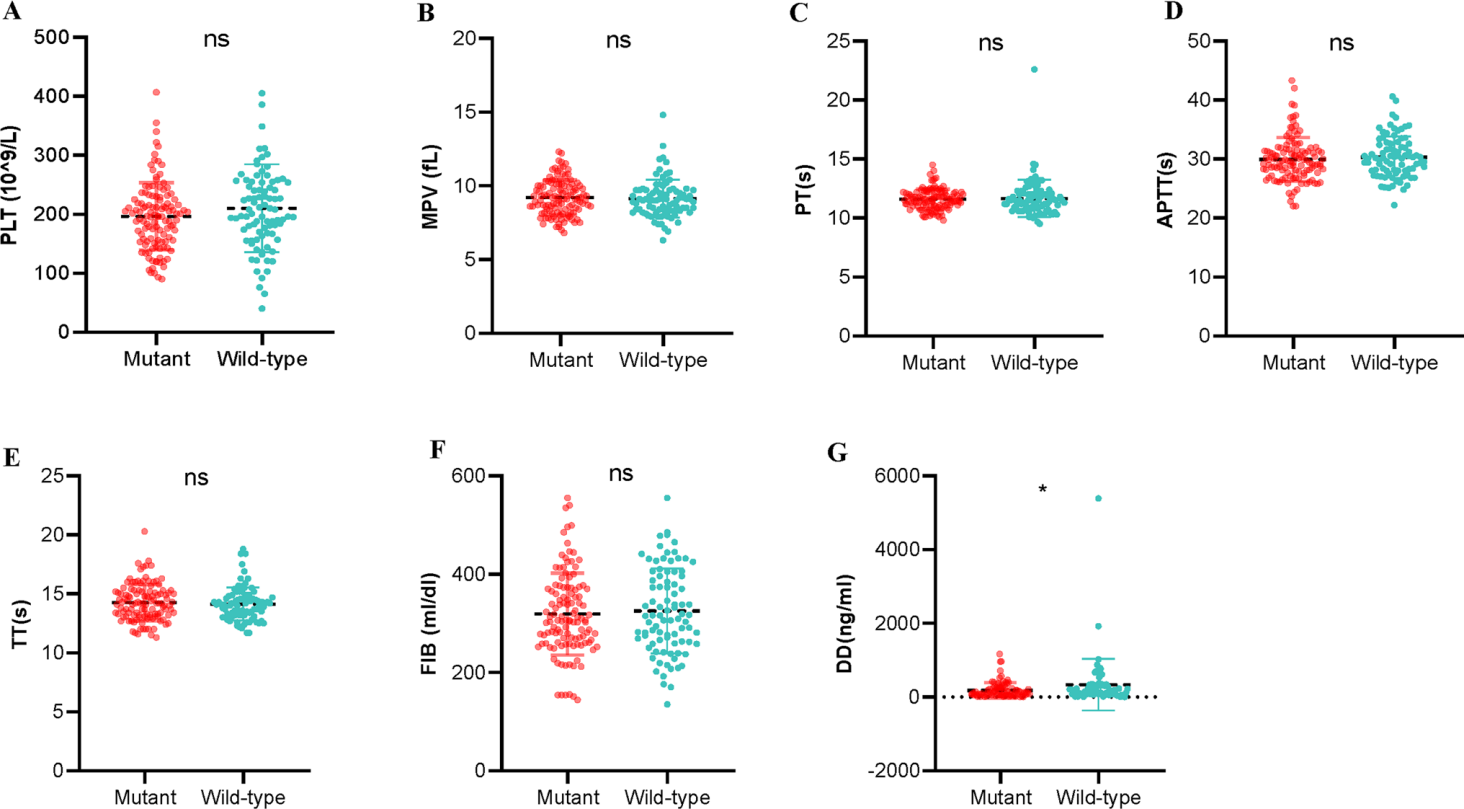
Mutations in TERT did no effects on PLT, MPV, PT, APTT, TT and FIB (**A**-**F**), but decreased levels of D-Dimer (**G**) based on the data from our cohort. ^*^P < 0.05, ns means *P* > 0.05 compared to TERT wild-type

A comparison was also made between patients with and without TERT mutations in terms of preoperative coagulation function. Platelet (PLT), mean platelet volume (MPV), prothrombin time (PT), activated partial thromboplastin time (APTT), and thrombin time (TT) is associated with hemostasis and secondary hemostasis. Fibrinogen (FIB), and D-dimer (D-D) are involved in fibrinolysis. The above items are the most commonly used clinical indicators to reflect hemostasis, coagulation, and fibrinolysis. We observed that the PLT, MPV, PT, APTT, TT, and FIB levels in TERT mutant and wild-type GBMs were comparable (Fig. 4A–F). However, TERT mutant GBMs were associated with lower levels of D-D than TERT wild-type GBMs, suggesting a systematically antithrombotic effect (Fig. 4G).

### TERT mutant GBM patients possessed shorter overall survival and poorer chemotherapy response

**Fig. 5:**
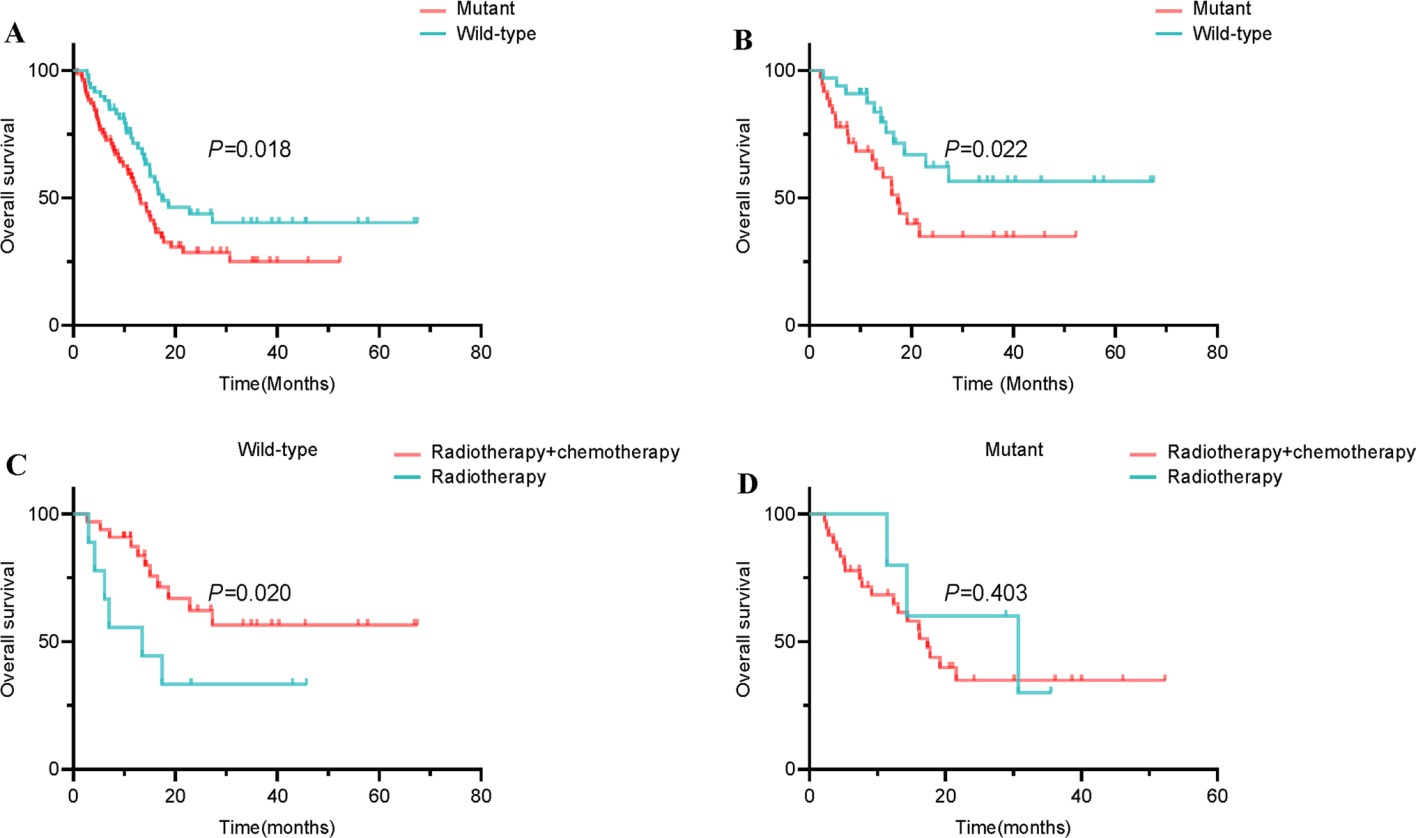
Effects of TERT mutations on the prognosis and chemotherapy of GBM patients based on the data from our cohort. **A** Mutations in TERT decreased overall survival in GBM patients compared to wild-type regardless of whether postoperative radiotherapy, chemotherapy, or radio-chemotherapy is received. **B** Mutations in TERT decreased overall survival in GBM patients compared to wild-type in those receiving standard STUPP chemo-radiotherapy treatment after surgery. **C** Chemotherapy plus radiotherapy improved overall survival in TERT wild-type GBM patients compared to radiotherapy alone. **D** Chemotherapy plus radiotherapy did not affect overall survival in TERT mutant GBM patients compared to radiotherapy alone

We further explored the effects of TERT on the prognoses of GBM patients. As shown in Fig. 5A, regardless of whether postoperative radiotherapy, chemotherapy, or radio-chemotherapy is received, the prognosis of TERT mutant patients was worse than that of wild-type patients. Besides, the overall survival in GBM patients with TERT mutations was also shorter than wild-type GBMs in those receiving standard STUPP chemo-radiotherapy treatment after surgery, which was validated by the data from the MSK cohort (Fig. 5B and Supplematary Fig. 3). Interestingly, chemotherapy (Temozolomide) combined with radiotherapy prolongs the overall survival of patients compared to radiotherapy alone in TERT wild-type GBMs, which was not observed in mutant GBMs (Fig. 5C, D).

### Effects of TERT mutations on immunotherapy response

**Fig. 6:**
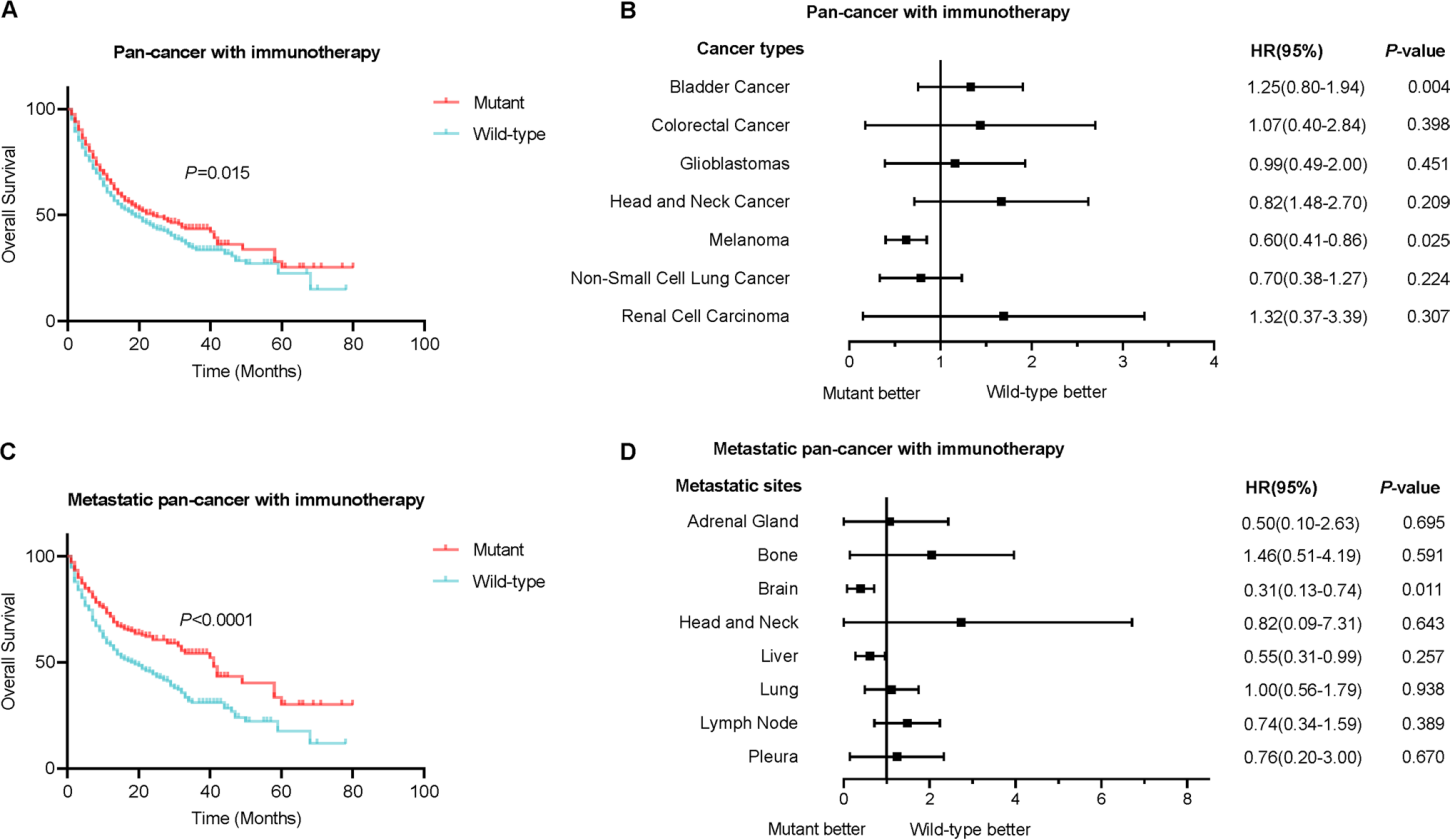
Differences in overall survival between TERT mutant and wild-type tumour patients receiving ICIs treatment. **A** Kaplan-Meier curves of patients with tumours receiving ICIs treatment in pan-cancer analysis. **B** Overall survival of patients with a given type of tumour receiving ICIs treatment. **C** Kaplan-Meier curves of patients with metastatic tumours receiving ICIs treatment in pan-cancer analysis. **D** Overall survival of patients with a given metastatic site of tumours receiving ICIs treatment

Finally, we explored the difference in immunotherapy response between TERT mutant and wild-type tumours. As shown in Fig. 6A, patients with TERT mutant tumours receiving ICI treatment exhibited longer survival times than those with TERT wild-type tumours. In a given type of tumour, TERT mutations were correlated with worse survival in bladder cancer, but better survival in Melanoma (Fig. 6B). However, the overall survival of TERT mutant and wild-type GBM patients were comparable. In addition, we found that patients with TERT mutant metastatic tumours receiving ICI therapy also presented better prognoses with respect to that in wild-type tumours (Fig. 6C). Interestingly, among these metastatic sites, patients with brain metastasis exhibited a better prognosis in TERT mutant tumours than that in wild-type tumours (Fig. 6D).

## Discussion

In the present study, we provided for the first time a comprehensive analysis of the differences in genetic alterations, clinicopathological characteristics, local and circulating immune microenvironment, chemotherapy and immunotherapy response, and overall survival between TERT mutant and wild-type GBMs. We found that the two types of GBMs possessed similar initial clinic symptoms, circulating immune microenvironment and immunotherapy response. With respect to that in TERT wild-type GBMs, patients with TERT mutations exhibited a lower frequency of TP53 mutations, higher levels of tumour-infiltrating neutrophils, worse chemotherapy response and poorer overall survival.

In the clinicopathological characteristics, we found that tumour location, sex distribution, diagnostic ages, and initial clinic symptoms were similar between TERT mutant and wild-type GBMs. Although there was an increasing trend in the incidence of epilepsy in GBM patients with TERT mutation, it did not reach statistical significance. In lower-grade gliomas, it has been demonstrated that IDH mutation, but not TERT mutation was associated with seizures [19]. These findings indicated that TERT mutation might be not correlated with the incidence of seizures in gliomas.

In the local immune microenvironment, we found that only levels of tumour-infiltrating neutrophils were increased following TERT mutation. However, in the circulating immune microenvironment, there was no difference in neutrophils between TERT mutant and wild-type GBMs. Neutrophils are the most abundant leukocytes in peripheral blood and are generally divided into N1 type (pro-tumour) and N2 type (anti-tumour) in cancers [20, 21]. Increased cell numbers of both circulating and tumour-infiltrating neutrophils were involved in an immunosuppressive environment and poor prognosis in tumours as well as gliomas [22–24]. In gliomas, tumour-infiltrating neutrophils could produce neutrophil extracellular traps to promote glioma progression via the HMGB1/RAGE/IL-8 axis[25]. Neutrophils could also increase treatment resistance in gliomas [26]. Therefore, high levels of local neutrophils in TERT mutant GBM patients might contribute to poor prognosis. Indeed, our present study found that patients with TERT mutant GBMs exhibited shorter survival times than that wild-type GBMs. Besides, high levels of neutrophils might also contribute to the failure of radiotherapy plus chemotherapy, because we found that TERT GBM patients could not benefit from the chemo-radiotherapy compared to radiotherapy alone. However, this hypothesis needs to be explored in the future.

In tumour immunotherapy, TERT mutations played diverse roles. For example, bladder cancer patients with TERT mutations exhibited shorter survival times, while melanoma patients with TERT mutations presented longer survival times. Consistent with our finding, two previous studies also found that melanoma patients bearing TERT mutation indeed benefited from immunotherapy [27, 28]. In contrast to bladder cancer, TERT mutation was also a favourable prognostic factor in urothelial carcinoma patients receiving ICI treatment [13]. Interestingly, there was no difference in overall survival in TERT mutant and wild-type GBM patients. This was the first report regarding the role of TERT mutations in the GBM immunotherapy cohort. Unlike GBMs, we found that brain metastasis with TERT mutations receiving ICI treatment exhibited longer survival times than those with TERT wild-type. Such opposite results might be mediated by the heterogeneity in the tumour immune environment between GBMs and brain metastasis. However, a large cohort of randomized controlled trials is needed to further validate this finding.

The present study has some limitations. First, this is a single-central study and large cohorts are needed for further validation. Second, the exact mechanisms of how mutant TERT alter the infiltration of neutrophils and chemotherapy response need further exploration.

## Conclusion

To summarize, this study comprehensively evaluated the differences in clinical characteristics and immune microenvironment between TERT mutant and wild-type GBMs. TERT mutations were associated with higher numbers of tumour-infiltrating neutrophils, a worse prognosis, and a reduced response to chemotherapy. All abbreviations and corresponding full names were presented in Table S1.

### Supplementary Information

Below is the link to the electronic supplementary material.

Supplementary Material 1

Supplementary Material 2

## Data Availability

The data used to support the findings of this study are available from the corresponding author upon request.
