# Supplementary material for: TERT mutations-associated alterations in clinical characteristics, immune environment and therapy response in glioblastomas
Source: Discov Oncol. 2023 Aug 11;14:148. doi: 10.1007/s12672-023-00760-w (PMC10421840; doi:10.1007/s12672-023-00760-w)
Supplement: Supplementary file 1 — Supplementary Material 1 [file 12672_2023_760_MOESM1_ESM.docx]

**Additional file 1: Figure S1 Effects of IDH mutations on overall survival of glioma patients based on the data from the MSK cohort**.

**Additional file 1: Figure S2 Effects of TERT mutations on the peripheral inflammatory markers based on peripheral blood data from our cohort.** ns means *P* >0.05 compared to TERT wild-type.

**Additional file 1: Figure S3 Mutations in TERT decreased overall survival in nGBM patients receiving chemo-radiotherapy based on the data from the MSK cohort.**
