# Supplementary material for: TERT mutations-associated alterations in clinical characteristics, immune environment and therapy response in glioblastomas
Source: Discov Oncol. 2023 Aug 11;14:148. doi: 10.1007/s12672-023-00760-w (PMC10421840; doi:10.1007/s12672-023-00760-w)
Supplement: Supplementary file 2 — Supplementary Material 2 [file 12672_2023_760_MOESM2_ESM.docx]

**Table S1 Abbreviations and full names**

| **Abbreviations** | **Full names** |
| --- | --- |
| **GBMs** | Glioblastomas |
| **TERT** | Telomerase reverse tranase |
| **IDH** | Isocitrate dehydrogenase |
| **CNS** | Central nervous system |
| **WHO** | World Health Organization |
| **IHC** | Immunohistochemistry |
| **NEUT** | Neutrophil |
| **LYMPH** | Lymphocyte |
| **MONO** | Monocyte |
| **EO** | Eosinophil |
| **BASO** | Basophil |
| **PLT** | Platelet |
| **MPV** | Mean platelet volume |
| **PT** | Prothrombin time |
| **APTT** | Activated partial thromboplastin time |
| **TT** | Thrombin time |
| **FIB** | Fibrinogen |
| **DD** | D-dimer |
| **NLR** | Neutrophil-to-lymphocyte ratio |
| **LMR** | Lymphocyte-to-monocyte ratio |
| **PLR** | Platelet-to-lymphocyte ratio |
| **SII** | Platelet*NLR |
| **ELR** | Eosinophil-to-lymphocyte ratio |
| **BLR** | Basophil-to-lymphocyte ratio |
